# Vat Photopolymerization of CeO_2_-Incorporated Hydrogel Scaffolds with Antimicrobial Efficacy

**DOI:** 10.3390/ma18051125

**Published:** 2025-03-02

**Authors:** Nelly Aimelyne Mpuhwe, Gyu-Nam Kim, Young-Hag Koh

**Affiliations:** 1 School of Biomedical Engineering, Korea University, Seoul 02841, Republic of Korea; mpuhwenelly@gmail.com (N.A.M.); gyunamkd@korea.ac.kr (G.-N.K.); 2 Interdisciplinary Program in Precision Public Health, Korea University, Seoul 02841, Republic of Korea

**Keywords:** hydrogel, gelatin methacryloyl, cerium oxide, vat photopolymerization, antimicrobial

## Abstract

We herein demonstrate the utility of gelatin methacryloyl (GelMA)/poly(ethylene glycol) diacrylate (PEGDA)–cerium oxide (CeO_2_) hydrogel inks for manufacturing hydrogel scaffolds with antimicrobial efficacy by vat photopolymerization. For uniform blending with GelMA/PEGDA hydrogels, CeO_2_ nanoparticles with a round shape were synthesized by the precipitation method coupled with calculation at 600 °C. In addition, they had highly crystalline phases and the desired chemical structures (oxidation states of Ce^3+^ and Ce^4+^) required for outstanding antimicrobial efficacy. A range of GelMA/PEGDA-CeO_2_ hydrogel scaffolds with different CeO_2_ contents (0% *w*/*v*, 0.1% *w*/*v*, 0.5% *w*/*v*, 1% *w*/*v*, and 5% *w*/*v* with respect to distilled water content) were manufactured. The photopolymerization behavior, mechanical properties, and biological properties (swelling and biodegradation behaviors) of hydrogel scaffolds were characterized to optimize the CeO_2_ content. GelMA/PEGDA-CeO_2_ hydrogel scaffolds produced with the highest CeO_2_ content (5% *w*/*v*) showed reasonable mechanical properties (compressive strength = 0.56 ± 0.09 MPa and compressive modulus = 0.19 ± 0.03 MPa), a high swelling ratio (1063.3 ± 10.9%), and the desired biodegradation rate (remaining weight after 28 days = 39.6 ± 2.3%). Furthermore, they showed outstanding antimicrobial efficacy (the number of colony-forming units = 76 ± 44.6 (×10^3^)). In addition, macroporous GelMA/PEGDA-CeO_2_ hydrogel scaffolds with tightly controlled porous structures could be manufactured by vat photopolymerization.

## 1. Introduction

Hydrogels have gained significant attention in tissue engineering due to their ability to mimic the extracellular matrix (ECM) of natural tissues when implanted into the human body [1,2,3,4,5]. In particular, they are comprised of a 3D network of semi-permeable, hydrophilic polymer chains, thus enabling them to absorb a large amount of water. This unique feature can provide favorable environments for the transport of body fluids, including blood, oxygen, nutrients, and growth factors essential to tissue regeneration [1,2]. In addition, hydrogels can offer high flexibility and mechanical properties similar to those of natural tissues [6,7]. Thus, they can be used for regenerating not only hard (e.g., bones) tissues but also soft (e.g., cartilages, skins, and nerves) tissues [3,4,5,8,9,10,11,12].

Among hydrogels, gelatin methacryloyl (GelMA) has demonstrated great promise since it can provide desired biological and mechanical functions when implanted into the human body, along with easy formulation into scaffolds [3,4,8,9,13,14,15,16,17]. More specifically, GelMA is a derivative of natural gelatin, thus offering biocompatible surfaces and sites for cell adhesion, which is one of the most crucial steps for new tissue formation, and reasonable biodegradability via enzymatic cleavage [3,4]. In addition, GelMA can be readily formulated into gel-like solids (hydrogels) through photo-crosslinking of methacrylamide and methacrylate side groups introduced by the modification of gelatin with methacrylic anhydride [3,4]. Thus, arbitrarily designed external and internal geometries specially designed for target tissues can be constructed using 3D printing techniques [13,14,15,16,17,18,19,20,21,22,23,24]. For example, three-dimensionally interconnected macroporous structures can facilitate new tissue formation by providing 3D pores for cell migration and large surfaces for cell adhesion, proliferation, and differentiation [16,18,19,20,21,22]. In addition, tubular and multichannelled structures can effectively guide nerve regeneration through unidirectionally aligned channels [23,24]. To this end, material extrusion (ME)- and vat photopolymerization (VP)-based 3D printing techniques have been widely utilized. The ME-based 3D printing technique can dispense GelMA-based solutions (inks) through fine nozzles and then deposit extruded filaments according to predetermined build paths with the assistance of photo-crosslinking for good shape retention [18,19,20]. However, this technique has limited dimensional accuracy owing to its material extrusion approach. On the other hand, the VP-based 3D printing technique can selectively photopolymerize thin layers of GelMA-based inks, for example, using high-resolution digital light processing (DLP) engines, thus allowing the construction of more sophisticated porous structures with high dimensionally accuracy [21,22,23,24].

To widen the clinical uses of GelMA-based hydrogel scaffolds, GelMA can be hybridized with other synthetic hydrogels, in order to enhance and/or tailor its mechanical properties and biological functions [20,22,23,24,25,26,27,28]. In particular, when hybridized with poly(ethylene glycol) diacrylate (PEGDA), a kind of PEG-derived synthetic hydrogel [5,10,11,29,30], the mechanical properties and biological functions (e.g., swelling and biodegradation behaviors) of GelMA/PEGDA hydrogel scaffolds can be enhanced and/or tailored by adjusting the GelMA/PEGDA ratio [20,22,23,24,26,27,28]. They can also be formulated into macroporous structures using the VP-based 3D printing technique [22,23,24]. Furthermore, to prevent infections and reduce postoperative complications, antimicrobial and antibacterial inorganic fillers can be incorporated into GelMA-based hydrogels, which can eradicate bacteria when implanted in the human body [31]. To this end, a variety of nanomaterials, including silver nanoparticles (AgNPs) [32,33,34], graphene oxide [35,36], zinc oxide (ZnO) [37,38], and cerium oxide (CeO_2_) [39,40], have been examined. In particular, cerium oxide can offer additional advantageous functions, especially for wound healing applications [41,42,43,44,45]. More specifically, its anti-inflammatory and antioxidant properties can remarkably reduce inflammatory responses and oxidative stress caused by excessive reactive oxygen species (ROS), respectively, thus alleviating damage to wound tissue. In addition, its angiogenesis property can facilitate the formation of new blood vessels from pre-existing vessels, which can accelerate wound healing by supplying oxygen and nutrients to the cells involved in tissue repair.

Thus, the main purpose of this study is to demonstrate the manufacturing of CeO_2_-incorporated GelMA/PEGDA hydrogel composite scaffolds with antimicrobial efficacy using VP-based 3D printing. In particular, to obtain homogeneous inks for VP-based 3D printing, several processing parameters were carefully controlled and optimized, including synthesis of CeO_2_ nanoparticles, sequence and temperature for blending of CeO_2_ solutions with PEGDA and GelMA solutions, inert dye content, and photo-crosslinking time. To optimize the CeO_2_ content, a range of GelMA/PEGDA-CeO_2_ inks with different CeO_2_ contents (0% *w*/*v*, 0.1% *w*/*v*, 0.5% *w*/*v*, 1% *w*/*v*, and 5% *w*/*v* with respect to distilled water content) and their rheological and photo-crosslinking behaviors were examined. The mechanical properties of printed hydrogels were examined by compressive strength tests. Their biological properties, including swelling behavior, biodegradation behavior, and antimicrobial efficacy, were examined.

## 2. Materials and Methods

### 2.1. Synthesis of CeO_2_ Nanoparticles

**Figure 1:**
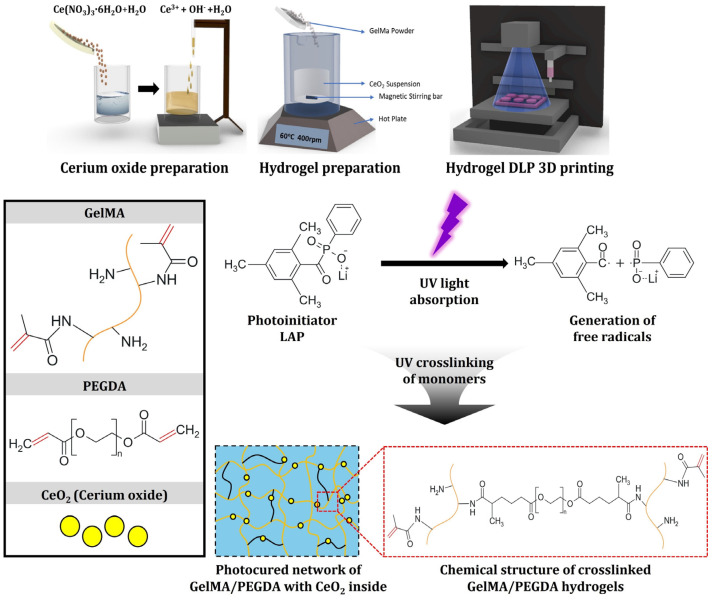
Schematic diagram showing the manufacturing of CeO_2_-incorporated GelMA/PEGDA hydrogel scaffolds by VP-based 3D printing using photo-crosslinking process.

A schematic diagram showing the manufacturing of CeO_2_-incorporated GelMA/PEGDA hydrogel scaffolds by VP-based 3D printing using the photo-crosslinking process is shown in Figure 1. Cerium oxide nanoparticles were synthesized by slightly modifying the precipitation method reported in the literature [46,47]. Briefly, 10.0 g of cerium nitrate hexahydrate (Ce(NO_3_)_3_·6H_2_O; 22.4 mmol, 1.0 eq.) supplied by Sigma Aldrich (Saint Louis, MO, USA) was completely dissolved in 115.0 cm^3^ of distilled water at 60 °C for 30 min by magnetic stirring. After which, 10.75 g of ammonia water (NH_4_OH (30%; 189.0 mmol, 8.4 eq.) supplied by Daejung Chemicals (Siheung-si, Gyeonggi-do, Republic of Korea) was added dropwise into the solution during magnetic stirring for 2 h, then cooled down to room temperature. Finally, the mixture was stirred for 24 h at room temperature to ensure a complete chemical reaction between reactants. Solid precipitates (Ce(OH)_4_·H_2_O) obtained were washed three times with distilled water and acetone in sequence, and then filtered. Precipitates were dried in a furnace (Ajeon Heating Industrial Co., Ltd., Namyangju-si, Gyeonggi-do, Republic of Korea) at 120 °C for 4 h, and then calcined at 600 °C for 2 h to obtain CeO_2_ nanoparticles.

### 2.2. Characterization of CeO_2_ Nanoparticles

The morphologies of synthesized CeO_2_ nanoparticles were examined using field emission scanning electron microscopy (FE-SEM; JSM-6701F, JEOL Techniques, Tokyo, Japan). Their crystalline structures and phases were analyzed by X-ray diffraction (P-XRD, Ultima III, Rigaku, Japan). X-ray photoelectron spectroscopy (XPS, X-Tool, Ulvac-Phi, Inc., Kanagawa, Japan) was also used to analyze the chemical compositions and oxidation states of CeO_2_ nanoparticles.

### 2.3. Preparation of GelMA/PEGDA-CeO_2_ Hydrogel Inks

GelMA was kindly supplied by KD Research Center (KD Research Center, Busan, Republic of Korea) and PEGDA monomer (Mn 575, Sigma Aldrich, Saint Louis, MO, USA) was used as received. A range of GelMA/PEGDA-CeO_2_ hydrogel inks with different CeO_2_ contents (0% *w*/*v*, 0.1% *w*/*v*, 0.5% *w*/*v*, 1% *w*/*v*, and 5% *w*/*v* with respect to distilled water content) were prepared (Table 1). The GelMA/PEGDA ratio was determined by considering our previous work [23] and preliminary tests. In addition, BYK-190 (BYK-Chemie Inc., Kempen, Germany) was used as the dispersant. As the photoinitiator, lithium phenyl (2,4,6-trimethylbenzoyl) phosphinate (LAP; Sigma Aldrich, Saint Louis, MO, USA) was employed particularly due to its low cytotoxicity and active wavelength for VP-based 3D printing [16]. The chemical structure of LAP and its working mechanism for the photo-crosslinking of GelMA/PEGDA hybrid hydrogels is shown in Figure 1. When excited by UV light (e.g., 405 nm), LAP can undergo cleavage and generate free radicals, which can initiate photo-crosslinking of photocurable side groups of GelMA and PEGDA, resulting in gel-like solids (hydrogels) tough chain propagation. Allura Red dye (Sigma Aldrich, Saint Louis, MO, USA) was used as the inert dye. To this end, predetermined amounts of CeO_2_ nanoparticle, dispersant, and inert dye were added into bottles containing zirconia balls used as the grinding and mixing medium. Distilled water was then added into mixtures and vigorously mixed by a planetary mixer (Hantech Co, Ltd., Hwaseong-si, Gyeonggi-do, Republic of Korea) for 30 min at 1000 rpm. After which, CeO_2_ suspensions obtained were sieved to remove the zirconia balls and transferred into glass beakers. CeO_2_ suspensions were placed at 60 °C to homogenously blend them with PEGDA and GelMA. Predetermined amounts of PEGDA monomers were added into CeO_2_ suspensions and mixed by magnetic stirring at 400 rpm for 1 h. After which, predetermined amounts of GelMA powders were gradually added into blends to ensure fast, complete dissolution. For VP-based 3D printing, the photoinitiator was added into GelMA/PEGDA-CeO_2_ hydrogel inks.

### 2.4. Photopolymerization Behaviors of GelMA/PEGDA-CeO_2_ Hydrogel Inks

To examine the utility of various GelMA/PEGDA-CeO_2_ hydrogel inks (CeO_2_ contents = 0% *w*/*v*, 0.1% *w*/*v*, 0.5% *w*/*v*, 1% *w*/*v*, and 5% *w*/*v*) for vat photopolymerization, their photopolymerization behaviors were evaluated using a photo-differential scanning calorimeter (Photo-DSC) (DSC4000; Perkin Elmer, Waltham, MA, USA). To this end, various hydrogel inks with different CeO_2_ contents (0% *w*/*v*, 0.1% *w*/*v*, 0.5% *w*/*v*, 1% *w*/*v*, and 5% *w*/*v*) were illuminated by UV light with a power of 20 mW/cm^2^ for 2 min. Heat flows generated by photopolymerization were monitored as a function of time and then used to compute the percent conversions of the hydrogel inks [48].

**Figure 2:**
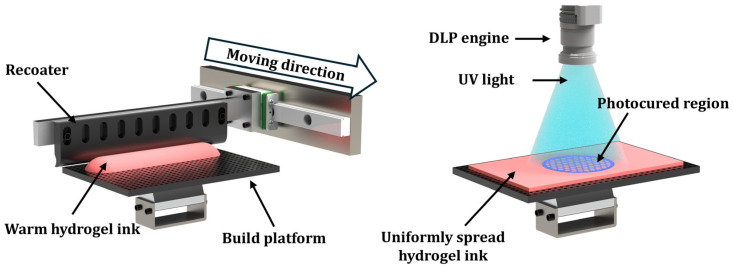
Schematic diagram of the DLP 3D printing technique with GelMA-based hydrogel.

In addition, to utilize our custom-built VP printer (Veltz3D, Incheon, Republic of Korea) for manufacturing hydrogel scaffolds, the photopolymerization behaviors of various hydrogel inks (CeO_2_ contents = 0% *w*/*v*, 0.1% *w*/*v*, 0.5% *w*/*v*, 1% *w*/*v*, and 5% *w*/*v*) were characterized. Warm hydrogel inks prepared at 60 °C were uniformly spread onto build platforms using a specially designed recoater (Figure 2) and then photopolymerized for 3 s using a digital light processing (DLP) engine illuminating UV light with a power of ~13.6 mW/cm^2^ at a peak wavelength of ~405 nm. After which, photopolymerized layers were carefully removed and their thicknesses were measured using a micrometer.

### 2.5. Vat Photopolymerization of GelMA/PEGDA-CeO_2_ Hydrogel Scaffolds

Several different types of GelMA/PEGDA-CeO_2_ hydrogel scaffolds (CeO_2_ contents = 0% *w*/*v*, 0.1% *w*/*v*, 0.5% *w*/*v*, 1% *w*/*v*, and 5% *w*/*v*) were manufactured using our custom-built VP printer. To evaluate the microstructures (e.g., hybridization behavior of GelMA/PEGDA with CeO_2_ nanoparticles), mechanical properties, and biological properties of hydrogel scaffolds, bulk samples without a 3D macroporous structure were manufactured using a layer thickness of 100 μm and UV illumination time of 3 s. In addition, as the model, 3D macroporous hydrogel scaffolds with a honeycomb-like structure were manufactured using the highest CeO_2_ content of 5% *w*/*v*.

### 2.6. Microstructure and Mechanical Properties of GelMA/PEGDA-CeO_2_ Hydrogel Scaffolds

The microstructures of various GelMA/PEGDA-CeO_2_ hydrogel scaffolds (CeO_2_ contents = 0% *w*/*v* and 5% *w*/*v*) were examined by FE-SEM. For these observations, samples were freeze dried to remove ice crystals formed via freezing of water. Their surfaces were then coated with gold using a magnetron sputter coater (108Auto, Cressington, UK) at 20 mA for 120 s under high vacuum, and then, FE-SEM images were taken at an acceleration voltage of 10 kV with a working distance of 8 mm. In addition, BSE (backscattered electron) images were taken during FE-SEM operations, in order to clarify the distribution of CeO_2_ nanoparticles in GelMA/PEGDA hybrids.

The mechanical properties of various GelMA/PEGDA-CeO_2_ hydrogel scaffolds (CeO_2_ contents = 0% *w*/*v*, 1% *w*/*v*, and 5% *w*/*v*) were evaluated by compressive strength tests using a universal testing machine (UTM; ST-1000, Salt Co., Ltd., Incheon, Republic of Korea). Cylindrical samples with a diameter of 12 mm and a height of 5 mm were compressed at a constant cross-head speed of 1 mm/min. Their compressive stresses as a function of strain were monitored, and were used to compute their compressive strengths and moduli. Five specimens for each composition were tested to assess the mean value and standard deviation.

### 2.7. Biological Properties of GelMA/PEGDA-CeO_2_ Hydrogel Scaffolds

The swelling behaviors, biodegradation behaviors, and antimicrobial efficacy of various GelMA/PEGDA-CeO_2_ hydrogel scaffolds (CeO_2_ contents = 0% *w*/*v*, 0.1% *w*/*v*, 0.5% *w*/*v*, 1% *w*/*v*, and 5% *w*/*v*) were examined. To this end, five cylindrical samples (12 mm in diameter and 5 mm in height) were manufactured for each composition.

For swelling tests, samples were immersed in phosphate-buffered saline (PBS) solutions (Sigma Aldrich, Saint Louis, MO, USA) at 37 °C for 3 days. The weights of swollen samples (*W_s_*) were measured by an electronic balance. After which, the swollen samples were fully freeze dried for 7 days, and their weights (*W_d_*) were measured. The swelling ratios of the hydrogel scaffolds were then computed, as follows:

Swelling ratio (%) = (W_s_ − W_d_)/W_d_ × 100 (1)

For biodegradation tests, samples fully swollen after immersion in a PBS solution for 3 days at 37 °C were soaked in collagenase solutions with a concentration of 10 mg/L (Sigma Aldrich, Saint Louis, MO, USA) up to 28 days. The weights of the samples (*W_b_*) were measured periodically with a time interval of 7 days. The biodegradability of samples was then computed as follows:

Biodegradability (%) = (W_s_ − W_b_)/W_s_ × 100 (2)

To assess the antimicrobial efficacy of CeO_2_ nanoparticles, GelMA/PEGDA-CeO_2_ hybrid scaffolds and GelMA/PEGDA-CeO_2_ hybrid scaffolds without CeO_2_ (0% *w*/*v*) and with the highest CeO_2_ content of 5% *w*/*v* were tested. As the model, *S. mutans* (NCTC 10449) cultured in Luria–Bertani broth (LB; BD Biosciences, Franklin Lakes, NJ, USA) were employed. In total, 1 mL of a bacterial suspension (1.1 × 10^6^ CFU/mL) was added to samples placed in 24-well plates and then incubated for 24 h. After which, the samples were gently washed four times with a phosphate-buffered saline solution (PBS; Thermo Fisher Scientific Inc., Waltham, MA, USA) to remove all the non-adherent bacteria. The attached bacteria were then harvested in a 3 mL Dulbecco’s phosphate-buffered saline (DPBS; Welgene, Gyeongsan-si, Gyeongsangbuk-do, Republic of Korea) solution by vortexing for 1 min. Then, 100 μL of harvested bacterial suspension for each composition was spread onto a solid agar plate and incubated for 24 h at 37 °C, and the total number of colonies were counted by ImageJ (Version 1.54g, U. S. National Institutes of Health, Bethesda, MD, USA).

### 2.8. Statistical Analysis

Before performing all parametric statistics, the normality of distributions was verified with the Shapiro–Wilk test. All data were expressed as the mean ± standard deviation. Statistical analysis was performed using a one-way analysis of variance (ANOVA) with Tukey’s post-hoc test using MATLAB (Release 2024b, The MathWorks, Inc., Natick, MA, USA). A *p*-value < 0.05 was considered statistically significant, which was graphically demonstrated with different superscripts within each figure.

## 3. Results

### 3.1. Characteristics of CeO_2_ Nanoparticles

To optimize the use of CeO_2_ as an antimicrobial agent in GelMA/PEGDA hydrogel scaffolds, CeO_2_ nanoparticles with specific physical and chemical characteristics were synthesized using the precipitation method reported in the literature [46,47]. However, we herein employed a relatively low temperature (600 °C) for calcination to avoid strong agglomeration between primary particles. Subsequently, the morphologies, crystalline phases, and chemical structures of the synthesized CeO_2_ nanoparticles were thoroughly characterized using various analytical tools.

**Figure 3:**
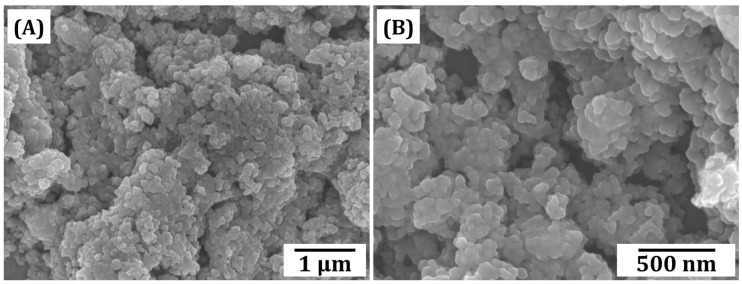
Representative FE-SEM images of synthesized CeO_2_ nanoparticles (**A**) at low and (**B**) at high magnifications.

The morphologies of synthesized CeO_2_ nanoparticles were examined by FE-SEM, and their representative FE-SEM images are shown in Figure 3. All primary particles showed a round shape with very small sizes in the range of ~80 nm–120 nm roughly estimated from the FE-SEM images. This is one of the most striking advantageous features of the precipitation method that can utilize a chemical reaction between ions dissolved in liquid mediums. Although the primary particles were partially bonded (Figure 3B), they were subsequently dispersed into nanoparticles during the preparation of CeO_2_ suspensions through a vigorous planetary mixing process.

**Figure 4:**
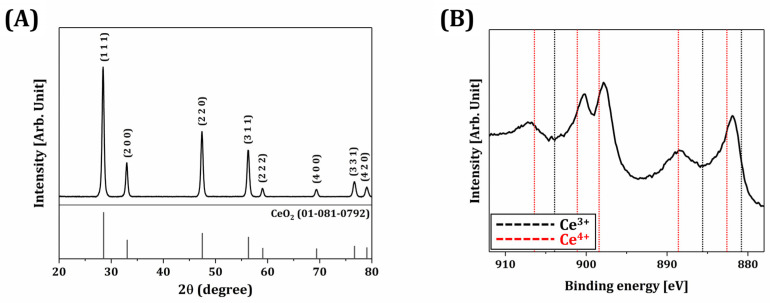
(**A**) XRD pattern and (**B**) XPS spectrum of synthesized CeO_2_ nanoparticles.

The crystalline phases of synthesized CeO_2_ nanoparticles were characterized by XRD, as shown in Figure 4A. A number of strong, sharp peaks were observed, suggesting that CeO_2_ nanoparticles could be highly crystallized after calcination even at a relatively low temperature of 600 °C. In addition, all peaks are well matched to those of CeO_2_ with a cubic fluorite structure (JCPDS card # 01-081-0792). No secondary phases were observed, indicating the high purity of synthesized CeO_2_ nanoparticles. Fundamentally, the antimicrobial efficacy of CeO_2_ is strongly affected by its oxidation states, often described as the Ce^3+^/Ce^4+^ ratio [43,44,49]. Thus, XPS was employed to clarify the oxidation states of Ce^3+^ and Ce^4+^, as shown in Figure 4B. Bind energies of 880.8 eV, 885.6 eV, and 903.9 eV are the binding energies of the Ce^3+^ oxidation state, and the other peaks correspond to the Ce^4+^ oxidation state. Note that the Ce^3+^ oxidation state of cerium oxide is well known to have anti-oxidation, anti-inflammatory, and anti-apoptosis abilities, and the extraordinary biological characteristics of cerium oxide due to the reduction of Ce^4+^ to Ce^3+^ [50,51,52].

### 3.2. Photopolymerization Behavior of GelMA/PEGDA-CeO_2_ Hydrogel Inks

**Figure 5:**
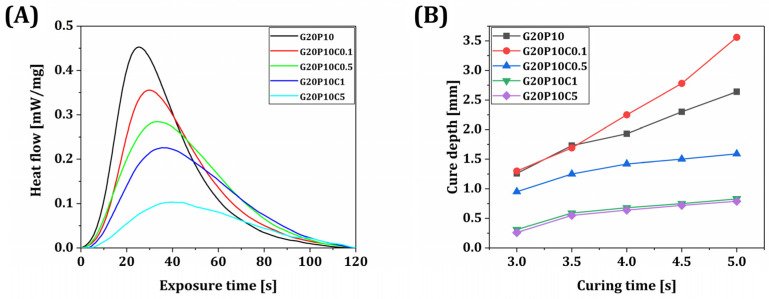
(**A**) Photo-DSC results and (**B**) cure depths obtained at 60 °C as a function of exposure time for photopolymerization from various GelMA/PEGDA-CeO_2_ hydrogel inks (CeO_2_ contents = 0% *w*/*v*, 0.1% *w*/*v*, 0.5% *w*/*v*, 1% *w*/*v*, and 5% *w*/*v*).

To fabricate GelMA/PEGDA-CeO_2_ hydrogel scaffolds with the high CeO_2_ contents necessary for effective antimicrobial functions, hydrogel inks should be efficiently photopolymerized using UV light powers compatible with VP printers. Thus, the influence of CeO_2_ addition on the photopolymerization behaviors of GelMA/PEGDA hydrogels was examined by photo-DSC, as shown in Figure 5A. All hydrogel inks showed one exothermic peak associated with photopolymerization of both GelMA and PEGDA (Figure 5A). However, the total enthalpy decreased with an increase in CeO_2_ content—16.7 J/g, 14.9 J/g, 14.4 J/g, 12.2 J/g, and 6.0 J/g for CeO_2_ contents of 0% *w*/*v*, 0.1% *w*/*v*, 0.5% *w*/*v*, 1% *w*/*v*, and 5% *w*/*v*, respectively. This trend can be attributed to the UV light scattering by CeO_2_ nanoparticles, which impedes the photopolymerization of GelMA/PEGDA hybrids. However, it should be noted that this concern would be mitigated by employing the thin layers of hybrid inks for layer-by-layer VP processes. In addition, all hybrid inks can be photopolymerized significantly within a reasonable period of time (~40 s), which would be applicable to conventional UV light engines used for VP printers.

To achieve strong interlayer bonding and high dimensional accuracy, optimization of UV exposure time is crucial for specific layer thicknesses, such as the 100 μm utilized in this study. To this end, the layers of various GelMA/PEGDA-CeO_2_ hydrogel inks (CeO_2_ contents = 0% *w*/*v*, 0.1% *w*/*v*, 0.5% *w*/*v*, 1% *w*/*v*, and 5% *w*/*v*) were photopolymerized using our custom-built VP printer. The cure depth defined by the thickness of the photopolymerized layer decreased with an increase in CeO_2_ content (Figure 5B), as expected from the photo-DSC results. However, the GelMA/PEGDA-CeO_2_ hydrogel with 0.1% *w*/*v* CeO_2_ content showed a higher cure depth than the GeIMA/PEGDA hydrogel. It is supposed that a small amount of CeO_2_ nanoparticles would slightly scatter UV light to local positions, thus resulting in thicker polymerized layers.

### 3.3. Microstructures of GelMA/PEGDA-CeO_2_ Hydrogel Scaffolds

**Figure 6:**
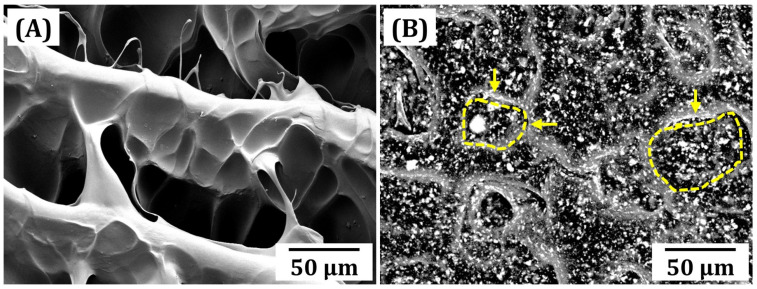
Representative FE-SEM images of GelMA/PEGDA-CeO_2_ hydrogel inks with various CeO_2_ contents of (**A**) 0% *w*/*v*, and (**B**) 5% *w*/*v*, where white contrast represents CeO_2_ nanoparticles. The yellow arrows indicate GelMA/PEGDA frameworks, and the inside marked by dashed line corresponds to pores.

To examine the utility of CeO_2_ nanoparticles synthesized in this study for blending with GelMA/PEGDA hydrogels, the microstructures of various GelMA/PEGDA-CeO_2_ hydrogel scaffolds manufactured by our custom-built VP printer were examined by FE-SEM. For these observations, hydrogel scaffolds were freeze dried to remove water. Figure 6A,B show the representative FE-SEM images of GelMA/PEGDA-CeO_2_ hydrogel scaffolds with different CeO_2_ contents (0% *w*/*v* and 5% *w*/*v*) for comparison purposes. Without CeO_2_ nanoparticles, the GelMA/PEGDA hybrid scaffold showed a highly porous structure comprised of relative dense GelMA/PEGDA frameworks (Figure 6A), where pores were created as the replica of ice crystals. To more clearly assess the distribution of CeO_2_ nanoparticles within GelMA/PEGDA frameworks, a BSE image was taken from the hydrogel scaffold with the highest CeO_2_ content (5% *w*/*v*) during FE-SEM operations (Figure 6B). It was observed that a number of CeO_2_ nanoparticles, appearing in the bright contrast, were uniformly distributed in GelMA/PEGDA frameworks.

It should be noted that as-manufactured hydrogel scaffolds can contain a large amount of water used as the solvent for GelMA powders. Thus, during freeze drying of hydrogel scaffolds required for FE-SEM observations, large ice crystals can be formed via the freezing of water and then sublimed, resulting in large pores. In addition, in the case of the BSE image (Figure 6B), it is reasonable to suppose that the yellow arrows indicate GelMA/PEGDA frameworks, and thus, the insides marked by dashed lines can be considered to be pores formed as the replica of ice crystals. This observation suggests that CeO_2_ nanoparticles can hinder the growth of ice crystals during freezing, thus resulting in a smaller pore size and slightly lower porosity. However, it should be noted that these pores can allow the transport of water, and thus, dried GelMA/PEGDA frameworks can reabsorb considerable amounts of water.

### 3.4. Mechanical Properties of GelMA/PEGDA-CeO_2_ Hydrogels

**Figure 7:**
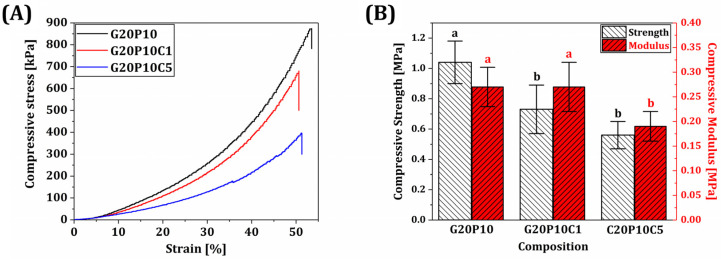
(**A**) Representative compressive stress–strain curves and (**B**) compressive strengths and moduli of various GelMA/PEGDA-CeO_2_ hydrogel inks (CeO_2_ contents = 0% *w*/*v*, 1% *w*/*v*, and 5% *w*/*v*). Different letters in each color represent statistical significance (*p*-value < 0.05).

To assess the structural stabilities of various GelMA/PEGDA-CeO_2_ hydrogel inks during tissue regeneration, the mechanical properties of various hydrogels were evaluated by compressive stress tests [53]. Note that GelMA/PEGDA and GelMA/PEGDA-CeO_2_ with high CeO_2_ contents (1% *w*/*v* and 5% *w*/*v*) were tested to clarify the influence of CeO_2_ on compressive strength. The representative compressive stresses as a function of strain are demonstrated in Figure 7A. All hydrogel scaffolds showed that stress increased almost linearly with an increase in strain and then dropped due to the eruption of water trapped. The compressive strengths and moduli of various hydrogel scaffolds computed from their stress–strain curves are demonstrated in Figure 7B. As the CeO_2_ content increased, the compressive strengths and moduli decreased. These reductions would presumably be due to the fact that interfaces between CeO_2_ nanoparticles and GelMA/PEGDA would act as crack origins during compression tests. However, if required, the mechanical properties of GelMA/PEGDA-CeO_2_ hydrogel scaffolds would be enhanced by creating strong interfacial bonding, for example, through surface modification of CeO_2_ nanoparticles with agents’ high affinity to GelMA/PEGDA hydrogels. It should be noted that GelMA/PEGDA-CeO_2_ hydrogel scaffolds with the highest CeO_2_ content (5% *w*/*v*) can provide reasonably high compressive strengths (0.56 ± 0.09 MPa) and moduli (0.19 ± 0.03 MPa) for soft tissue regeneration (e.g., wound healing) [54,55].

### 3.5. Swelling and Biodegradation Behaviors of GelMA/PEGDA-CeO_2_ Hydrogel Scaffolds

**Figure 8:**
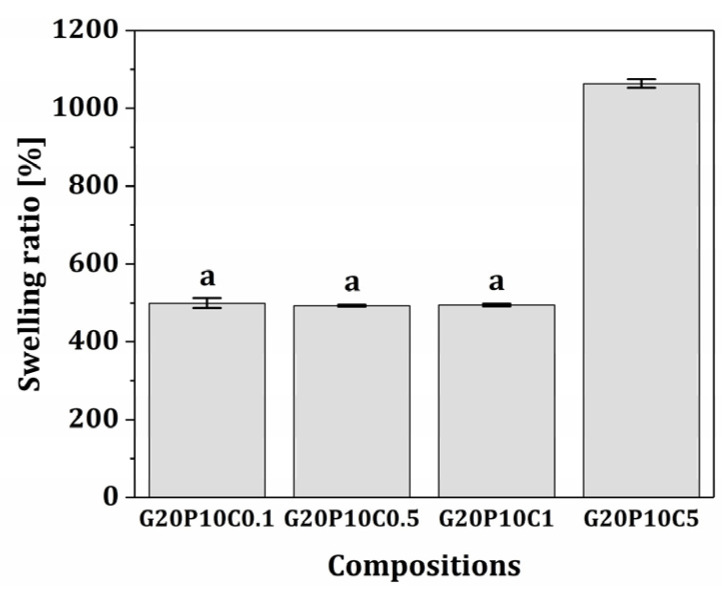
Swelling ratios of various GelMA/PEGDA-CeO_2_ hydrogel scaffolds (CeO_2_ contents = 0.1% *w*/*v*, 0.5% *w*/*v*, 1% *w*/*v*, and 5% *w*/*v*). Same letters (a) represent no statistical significance (*p*-value > 0.05).

To assess the structural integrity of GelMA/PEGDA-CeO_2_ hydrogel scaffolds, their swelling and biodegradation behaviors were examined. The swelling behaviors of various hydrogel scaffolds (CeO_2_ contents = 0.1% *w*/*v*, 0.5% *w*/*v*, 1% *w*/*v*, and 5% *w*/*v*) were examined by measuring their water absorption capabilities in PBS solutions. When the relatively low CeO_2_ contents were used, all hydrogel scaffolds showed similar swelling ratios—499.3 ± 12.7%, 492.9 ± 2.6%, and 494.7 ± 3.5% for CeO_2_ contents of 0.1% *w*/*v*, 0.5% *w*/*v*, and 1% *w*/*v*, respectively (Figure 8). This finding suggests that the GelMA/PEGDA hydrogel networks produced by our VP printer are permeable, thus absorbing a considerable amount of body fluids when implanted into the human body. However, the hybrid scaffolds with the highest CeO_2_ content (5% *w*/*v*) showed much higher swelling ratios (1063.3 ± 10.9%) than other scaffolds. This lies in the fact that CeO_2_ nanoparticles would hinder the crosslinking of GelMA/PEGDA hydrogel networks during photopolymerization by our VP printer, thus offering the higher water absorption capability.

**Figure 9:**
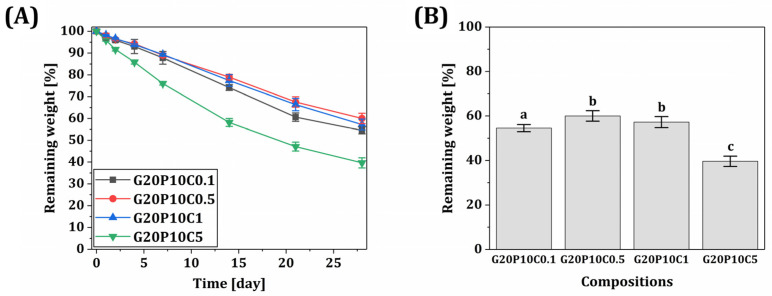
(**A**) Remaining weights of various GelMA/PEGDA-CeO_2_ hydrogel scaffolds (CeO_2_ contents = 0.1% *w*/*v*, 0.5% *w*/*v*, 1% *w*/*v*, and 5% *w*/*v*) during biodegradation in PBS-collagenase solutions as a function of time and (**B**) remaining weights after 28 days. Different letters represent statistical significance (*p*-value < 0.05).

The biodegradation behaviors of various hydrogel scaffolds were examined by measuring their mass losses in PBS-collagenase solutions (Figure 9A). The hybrid scaffolds with relatively low CeO_2_ contents showed similar weights remaining—54.5 ± 1.6%, 60.0 ± 2.4%, and 57.2 ± 2.5% for CeO_2_ contents of 0.1% *w*/*v*, 0.5% *w*/*v*, and 1% *w*/*v*, respectively (Figure 9B). On the other hand, when the highest CeO_2_ content (5% *w*/*v*) was used, notably lower remaining weights (39.6 ± 2.3%) were observed simply due to a reduction in the fraction of biodegradable GelMA/PEGDA hydrogel networks.

### 3.6. Antimicrobial Efficacy of GelMA/PEGDA-CeO_2_ Hydrogel Scaffolds

**Figure 10:**
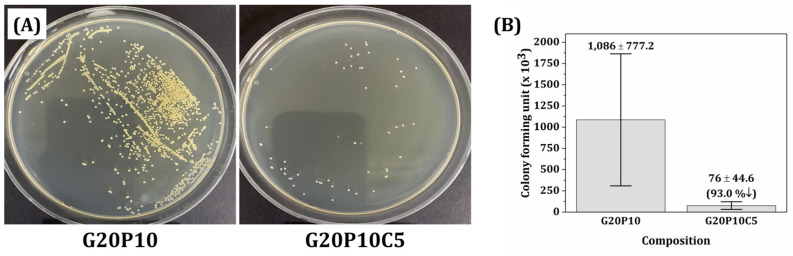
(**A**) Optical images of *S. mutans* adhered to GelMA/PEGDA and GelMA/PEGDA-CeO_2_ hydrogel scaffolds (CeO_2_ contents = 5% *w*/*v*) and (**B**) the numbers of colony-forming units.

For clinical uses for wound healing, hydrogel scaffolds are preferred to have antimicrobial efficacy to avoid the use of additional antibiotics [56]. By considering the mechanical properties, swelling behavior, and biodegradation behavior of GelMA/PEGDA-CeO_2_ hydrogel scaffolds, the highest CeO_2_ content of 5% *w*/*v* was employed for evaluating antimicrobial efficacy. For comparison purposes, the GelMA/PEGDA hydrogel scaffold without CeO_2_ addition was also tested. It was observed that the number of *S. mutans* used as the model bacterium decreased remarkably with CeO_2_ addition (Figure 10A). The numbers of colony-forming units were 1086 ± 777 (×10^3^) and 76 ± 44.6 (×10^3^) for the GelMA/PEGDA and GelMA/PEGDA-CeO_2_ hybrid scaffolds with a CeO_2_ cont I confirm ent of 5% *w*/*v*, respectively.

This finding demonstrates that CeO_2_ nanoparticles synthesized in this study can effectively act as the antimicrobial agent by negligibly altering the biological and mechanical functions of GelMA/PEGDA hydrogel scaffolds [57,58,59]. More specifically, when the reasonably high CeO_2_ contents are employed, a fraction of CeO_2_ nanoparticles can be located at and near the surfaces of GelMA/PEGDA frameworks, and the fraction can increase due to the swelling and biodegradation of GelMA/PEGDA frameworks, thus offering long-term antimicrobial efficacy.

### 3.7. VP of Macroporous GelMA/PEGDA-CeO_2_ Hydrogel Scaffolds

**Figure 11:**
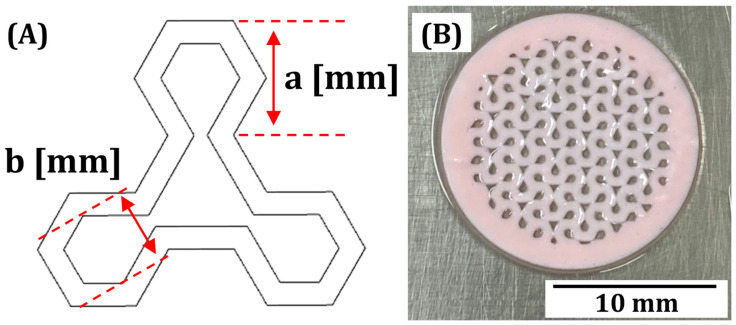
(**A**) A unit cell used to manufacture honeycomb-inspired scaffolds and (**B**) optical image of the GelMA/PEGDA-CeO_2_ hydrogel scaffold manufactured by our VP printer. Letter ‘a’ represents the channel size, and letter ‘b’ represents wall thickness of honeycomb-inspired structure.

To demonstrate the utility of GelMA/PEGDA-CeO_2_ hydrogel inks for VP, macroporous hydrogel scaffolds were manufactured by our custom-built VP printer. As the model, a honeycomb-inspired structure with a channel size (a) of 1 mm and wall thickness (b) of 0.5 mm was designed (Figure 11A). This unique macroporous structure can provide large surface areas for new tissue formation and pore sizes for a favorable VP process. Owing to the carefully optimized processing parameters for VP printing, including a layer thickness of 100 μm and UV illumination time of 3 s, allowed for manufacturing of macroporous GelMA/PEGDA-CeO_2_ hydrogel scaffolds even with the highest CeO_2_ content of 5% *w*/*v*, the manufactured hydrogel scaffolds showed highly controlled porous structures with a channel size of ~0.8 mm and wall thickness of ~0.6 mm. This finding suggests that our newly formulated GelMA/PEGDA-CeO_2_ hydrogel inks can be used to manufacture a variety of hydrogel scaffolds for tissue regeneration, particularly with antimicrobial efficacy using VP.

## 4. Conclusions

GelMa/PEGDA-CeO_2_ hydrogel inks were developed for vat photopolymerization of hydrogel scaffolds with antimicrobial efficacy. The CeO_2_ nanoparticles synthesized by the precipitation method could be uniformly blended with GelMA/PEGDA hydrogels owing to their round shape. In addition, the desired chemical structures (oxidation states of Ce^3+^ and Ce^4+^) and highly crystalline phases of CeO_2_ nanoparticles enabled GelMA/PEGDA-CeO_2_ hydrogel scaffolds (CeO_2_ content = 5% *w*/*v*) to have outstanding antimicrobial efficacy while maintaining reasonable compressive strengths and moduli, a high swelling ratio, and the desired biodegradation rate. In addition, hydrogel inks could be successfully utilized for manufacturing macroporous hydrogel scaffolds with tightly controlled porous structures by vat photopolymerization at 60 °C.

## Figures and Tables

**Table 1:** Components used to prepare GelMA/PEGDA-CeO_2_ hydrogel inks for vat photopolymerization and their fractions (% *w*/*v*) calculated by considering the volume of distilled water used as the solvent for GelMA.

Composition	GelMA (% *w*/*v*)	PEGDA (% *w*/*v*)	CeO_2_ (% *w*/*v*)	BYK-190 (% *w*/*v*)	LAP (% *w*/*v*)	Inert Dye (% *w*/*v*)
G20P10	20	10	0	0	0.2	0.01
G20P10C0.1			0.1	0.1		
G20P10C0.5			0.5	0.5		
G20P10C1			1	1		
G20P10C5			5	5		

## Data Availability

The original contributions presented in this study are included in the article. Further inquiries can be directed to the corresponding author.
